# The Association Between COVID-19-Related Persistent Symptoms, Psychological Flexibility, and General Mental Health Among People With and Without Persistent Pain in the UK

**DOI:** 10.3390/clinpract15070119

**Published:** 2025-06-25

**Authors:** Lin Yu, Lance M. McCracken

**Affiliations:** 1 Department of Psychology, Middlesex University, London NW4 4BT, UK; 2 Department of Psychology, Uppsala University, 752 37 Uppsala, Sweden; lance.mccracken@psyk.uu.se

**Keywords:** “long COVID”, pain, persistent symptoms, psychological flexibility, mental health

## Abstract

**Objectives:** Persistent symptoms following COVID-19 may adversely impact the general mental health of people with chronic pain, and psychological flexibility may buffer these impacts. However, it remains unclear whether such lasting implications of COVID-19 differ between people with and without chronic pain. This study investigated the relationships between persistent symptoms post-COVID-19, psychological flexibility, and general mental health among people with and without persistent pain during the COVID-19 pandemic in the UK. **Methods:** A total of 204 adults living in the UK were recruited via social media and completed an online survey, including measures of persistent symptoms, depression (Patient Health Questionnaire-9), anxiety (General Anxiety Disorder-7), insomnia (the Insomnia Severity Index), and psychological flexibility (the Multidimensional Psychological Flexibility Inventory), and were included in the analyses. **Results:** Participants with persistent pain (n = 70) experienced more-persistent symptoms, poorer general mental health, and a higher level of psychological inflexibility compared with participants without persistent pain (n = 133). Overall, the relationships between persistent physical symptoms, general mental health, and psychological (in)flexibility showed similar patterns in the two groups. Participants with more-persistent physical symptoms experienced significantly poorer general mental health. Furthermore, people with higher levels of psychological inflexibility reported worse general mental health. There was little evidence that psychological (in)flexibility could “buffer” the association between persistent physical symptoms and general mental health. **Conclusions:** People with chronic pain appear more vulnerable to persistent symptoms and reduced general mental health compared with people without pain. Treatments that reduce psychological inflexibility, such as ACT, may improve outcomes for people with persistent symptoms post-COVID-19.

## 1. Introduction

The impact of the COVID-19 pandemic on mental health is well documented [1]. This impact appears particularly severe among people with chronic pain, demonstrated by elevated levels of depression, anxiety, and insomnia during the pandemic [2,3,4]. In a large Canadian study including over three thousand individuals living with chronic pain, almost half of the individuals reported moderate or severe levels of psychological distress during the pandemic [3]. In an online survey of people with chronic pain early into the pandemic, about 80% reported moderate to severe depressive symptoms [2]. Nevertheless, COVID-19 remains a global challenge and continues to have a lasting detrimental impact.

As of 2 January 2023, two million people living in private households in the UK were estimated to be experiencing self-reported long-term effects of COVID-19, often described as “long COVID”, with almost 80% of them reporting limited day-to-day activities due to these continuing symptoms [5]. There is also evidence supporting the association between persistent COVID-19 symptoms and problems with general mental health [6,7]. The implication of “long COVID” may be particularly relevant for people with chronic pain [8]. In a study with over 700 individuals hospitalised for COVID-19, fifty percent of the individuals with pre-existing musculoskeletal pain experienced a worsening of their symptoms after COVID-19 [9].

Despite the prevalence of “long COVID” and its impact on health and functioning, “long COVID” is often undiagnosed due to the limited understanding of long COVID, restricted healthcare resources, and the lack of diagnosis of initial COVID-19 infection [10,11]. Nevertheless, regardless of a “long COVID” diagnosis, challenges that come with living with these persistent symptoms are probably not unlikely.

A set of psychological processes related to psychological flexibility (PF) may mitigate the impact of COVID-19 [4,12,13,14]. Psychological flexibility includes six processes: acceptance, cognitive defusion, present awareness, self-as-context, values, and committed action [15]. In essence, these processes reflect a set of skills to “recognize and adapt to various situational demands; shift mindsets or behavioural repertoires when these strategies compromise personal or social functioning; maintain balance among important life domains; and be aware, open, and committed to behaviours that are congruent with deeply held values” [16]. The pathological processes related to psychological flexibility are called psychological inflexibility.

Preliminary evidence suggests a buffering role of PF-related processes in the impact of COVID-19 on people with chronic pain. In a UK study of people with chronic pain [2], PF processes, including pain acceptance, committed action, and self-as-context, were found to mitigate the influence of the fear of COVID-19 and COVID-19-related avoidance in pain-related disability, mood, daily functioning, or depression. In a Swedish study in the context of COVID-19, PF processes were found to be associated with depression, anxiety, and insomnia in people with persistent pain [4]. In fact, a PF-based psychological treatment, called acceptance and commitment therapy (ACT) [15], has been applied to help people affected by COVID-19-induced stress and has shown promising results [17].

Despite the accumulating evidence for the lasting impact of COVID-19, including “long COVID”-related persistent symptoms, the association between persistent symptoms and general mental health, and its potential moderating factors, has rarely been explored. Furthermore, people with persistent pain and those without pain have not been directly compared, which has limited our understanding of the unique lasting challenge of COVID-19 for people with chronic pain. The aim of this study was to directly compare people with persistent pain with those without pain regarding the implications of persistent symptoms and the potential protective role of psychological flexibility in a UK community sample. This included three objectives:

1. To investigate whether participants with and without persistent pain differed in total persistent symptoms, general mental health, psychological flexibility, and psychological inflexibility. It was hypothesised that participants with persistent pain would report poorer mental health and lower psychological flexibility/higher psychological inflexibility.
2. To investigate the correlations between total persistent physical symptoms, psychological (in)flexibility, and general mental health among participants with and without pain respectively. It was hypothesised that a higher number of persistent physical symptoms would be correlated with poorer general mental health, and a higher level of psychological flexibility/lower level of psychological inflexibility would be correlated with better mental health. The correlation between psychological (in)flexibility and persistent physical symptoms was explored.
3. To explore if psychological (in)flexibility moderated the relationship between total persistent physical symptoms and general mental health among participants with and without pain respectively.

## 2. Methods

### 2.1. Study Design

This study used secondary analyses of data from a cross-sectional online survey study on persistent symptoms and general mental health during the COVID-19 pandemic in the UK, with a cross-sectional, correlational design.

### 2.2. Procedures

Ethical approval was obtained for this study from the Psychology Research Ethics Committee of the first author’s affiliated institution (Middlesex University; reference number: 18353 on 27 July 2021). Informed consent was obtained from all participants.

An advertisement for this study that included the weblink for the online survey was distributed on online social media platforms. Potential participants accessed the online survey via the weblink to review the participant information sheet. Potential participants were then asked to provide their consent to participate. Those who consented to participate in this study were directed to the screening page of the survey, and those who did not would exit the survey automatically. On the screen page, potential participants answered questions to assess their eligibility. People who were eligible proceeded to complete the survey (adults living in the UK), and those who were not would automatically exit the survey. At the end of the survey, participants were shown the debriefing, where information about supporting resources was included.

### 2.3. Participants

**Figure 1:**
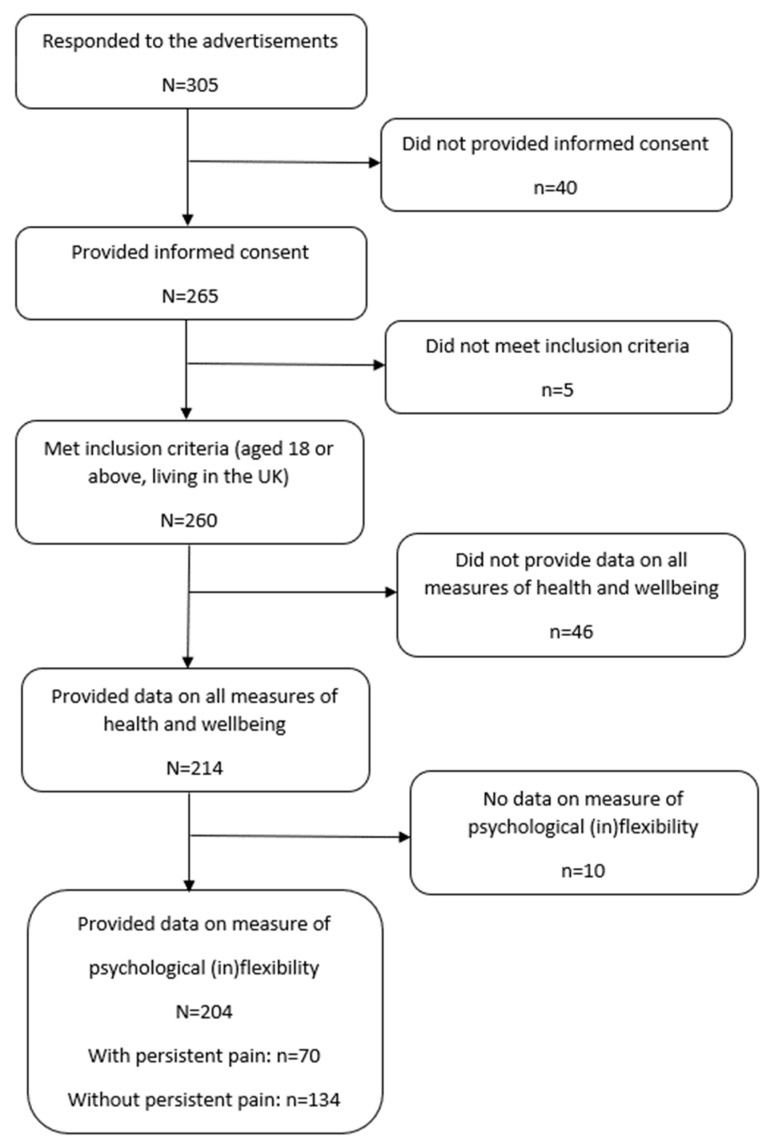
Flowchart for the recruitment process.

A total of 204 adults (aged 18 or above) living in the UK were included in this study. Figure 1 shows the recruitment process. A total of 305 people responded to the advertisement; 265 potential participants provided consent, among which 260 were eligible. Out of these 260 participants, 204 provided data for the study variables (70 with persistent pain and 134 without persistent pain). Participants were asked to indicate if they had persistent pain (pain most days during the last three months or longer). Table 1 shows the demographic information for participants with persistent pain and those without persistent pain. Participants with persistent pain and those without pain generally demonstrated similar profiles, with more men (60% and 51.9%, respectively), the majority being white (81.4% and 85.7%, respectively) and with an average age of about 40 years old (42.28 and 37.86, respectively), with had an average of about 15 years of education (14.71 and 15.44, respectively). In both groups, about half of the participants were working full-time, and about half reported an average economic status.

### 2.4. Measures

#### 2.4.1. Assessment of Persistent Symptoms

The assessment of persistent symptoms was adopted from a Swedish study on long COVID symptoms and general mental health [6]. Current (having persisted for at least six weeks) persistent symptoms were assessed with 26 items. The responses were yes or no. The total number of persistent symptoms was calculated by adding up the number of symptoms that were answered “yes” to. The total number of persistent physical symptoms was also calculated by adding up the number of symptoms that were answered “yes” to, except for depression, anxiety, sleep difficulties, decreased daily functioning, and decreased life quality.

#### 2.4.2. Patient Health Questionnaire-9 (PHQ-9)

The PHQ-9 is a ten-item self-report assessment for depression severity [18], with nine items assessing symptoms of depression, and the last item assessing the impact of depression. The total score of the first nine items indicates the severity of depression. In this sample, the PHQ-9 demonstrated excellent internal consistency, with Cronbach’s α = 0.91.

#### 2.4.3. General Anxiety Disorder-7 (GAD-7)

The GAD-7 is a seven-item self-report measure designed for screening GAD and assessing its severity [19]. In the current sample, the GAD-7 demonstrated excellent internal consistency, with Cronbach’s α = 0.93.

#### 2.4.4. Insomnia Severity Index (ISI)

The ISI is a seven-item self-report questionnaire assessing the nature, severity, and impact of insomnia [20,21]. In the current sample, the ISI demonstrated excellent internal consistency, with Cronbach’s α = 0.93.

#### 2.4.5. Multidimensional Psychological Flexibility Inventory (MPFI)

The MPFI is a self-report questionnaire that comprehensively assesses psychological flexibility. It includes sixty items reflecting the 12 dimensions/subscales of psychological flexibility and psychological inflexibility [22]. The averages of the six flexibility/inflexibility subscales create a composite representing global flexibility/inflexibility. In the current sample, all MPFI subscales demonstrated excellent internal consistency, with Cronbach’s α = 0.91–94, except for the acceptance subscale, which demonstrated good internal consistency, with Cronbach’s α = 0.83. The global psychological flexibility scale and the global psychological inflexibility scale demonstrated excellent internal consistency, with Cronbach’s α = 0.97 and 0.96, respectively.

### 2.5. Statistical Analysis

It was reported in the primary analyses of the data [23] that these background variables were generally not associated with the psychological (in)flexibility or the measures of mental health. Therefore, given the exploratory nature of the analyses, participants with and without persistent pain were not formally compared in terms of these background variables, and these variables were not controlled for in the main analyses.

Independent-samples *t*-tests were conducted to compare participants with and without persistent pain (IVs) in terms of total persistent symptoms, general mental health, and psychological (in)flexibility (DVs). Pearson’s correlations were conducted to investigate the correlations among psychological (in)flexibility, total persistent physical symptoms, and health and well-being. Moderated regression analyses, with total persistent symptoms and psychological (in)flexibility as the IVs, and measures of general mental health as the DVs, were conducted for participants with and without persistent pain separately to investigate if psychological (in)flexibility moderated the relationships between total persistent physical symptoms and health and well-being. Missing data were deleted pairwise.

Histograms and Q–Q plots were examined for all variables included in the *t*-tests and the moderated regressions in the total sample and each subgroup, respectively. The data for all variables were generally normally distributed, and there was no concern for the violation of the normality assumption.

## 3. Results

### 3.1. T-Tests (Study Objective 1)

Table 2 shows the descriptive statistics for the general mental health variables, PF variables, and total COVID-19 symptoms, and the *t*-test results.

Participants with persistent pain showed significantly higher levels of depressive symptoms, anxiety, and insomnia, higher levels of psychological inflexibility compared with people without persistent pain, and significantly more-persistent symptoms compared with participants without persistent pain.

### 3.2. Correlations (Study Objective 2)

Table 3 shows the correlations between psychological flexibility and psychological inflexibility, general mental health, and total persistent physical symptoms (excluding depression, anxiety, sleep difficulties, decreased daily functioning, and decreased life quality) among people with persistent pain and those without pain.

Overall, similar patterns of correlations were observed in participants with persistent pain and those without pain. Persistent physical symptoms significantly correlated with all measures of general mental health across the two sub-samples, and more strongly among people without persistent pain. Psychological flexibility and psychological inflexibility generally did not correlate with persistent physical symptoms. Psychological inflexibility showed stronger correlations with measures of general mental health compared with psychological flexibility.

### 3.3. Moderation (Study Objective 3)

#### 3.3.1. Moderation Analyses Among Participants with Persistent Pain

There was a significant interaction between total persistent physical symptoms and psychological flexibility for depression (*F* (1, 66) = 6.87; *p* < 0.05) but not psychological inflexibility (*F* (1, 62) = 0.39; *p* = 0.53). When Bonferroni correction was applied (critical a = 0.025), the interaction between total persistent physical symptoms and psychological flexibility remained significant.

Table 4 shows the conditional effect of total persistent physical symptoms on depression at levels of psychological flexibility. Total persistent physical symptoms were significantly associated with depression among participants with medium (M = 3.4) and high (M = 4.22) levels of psychological flexibility, but not participants with low levels of psychological flexibility (M = 2.46).

There was no significant interaction between total persistent physical symptoms and psychological flexibility (*F* (1, 66) = 3.19; *p* = 0.08) or psychological inflexibility (*F* (1, 62) = 0.04; *p* = 0.85) for anxiety.

There was no significant interaction between total persistent physical symptoms and either psychological flexibility (*F* (1, 66) = 3.42; *p* = 0.07) or psychological inflexibility (*F* (1, 62) = 0.25; *p* = 0.62) for insomnia.

#### 3.3.2. Moderation Analyses Among Participants Without Persistent Pain

There was no significant interaction between total persistent physical symptoms and either psychological flexibility (*F* (1, 129) = 0.01; *p* = 0.92) or psychological inflexibility (*F* (1, 121) = 0.35; *p* = 0.56) for depression.

There was no significant interaction between total persistent physical symptoms and psychological flexibility for anxiety (*F* (1, 129) = 0.76; *p* = 0.39) or psychological inflexibility (*F* (1, 121) = 1.87; *p* = 0.17).

There was no significant interaction between total persistent physical symptoms and either psychological flexibility (*F* (1, 129) = 0.47; *p* = 0.49) or psychological inflexibility (*F* (1, 121) = 1.97; *p* = 0.16) for insomnia.

## 4. Discussion

### 4.1. Discussion of Study Results

The experience of pain appears to be intertwined with the experience of “long COVID”. In our study, up to 30% of the participants reported various painful COVID-19-related persistent symptoms. The aim of this study was to directly compare people with persistent pain with those without pain based on the implications of persistent symptoms and the potential protective role of psychological flexibility in a UK community sample.

We found that participants with persistent pain experienced more persistent symptoms, poorer general mental health, and a higher level of psychological inflexibility compared with participants without persistent pain (study objective 1). Overall, the relationships between persistent physical symptoms, general mental health, and psychological (in)flexibility were consistent across participants with or without persistent pain. Participants with more persistent physical symptoms experienced significantly poorer general mental health. However, their persistent symptoms burdens were not associated with their levels of psychological flexibility or psychological inflexibility. Furthermore, people with higher levels of psychological inflexibility reported worse general mental health (study objective 2). There was little evidence that psychological (in)flexibility could “buffer” the association between persistent physical symptoms and general mental health (study objective 3).

To our knowledge, this is the first study directly comparing people with persistent pain with those without pain regarding persistent symptoms and general mental health in the context of COVID-19. This is also the first study investigating the moderating role of psychological flexibility in the relationship between persistent symptoms and general mental health in the UK, and the first comparing this relationship in people with persistent pain and those without pain.

Unsurprisingly, participants with persistent pain reported more persistent symptoms than those without. This may be because some participants reporting persistent pain had pre-existing chronic pain conditions, thus reporting pre-existing persistent symptoms that are commonly co-morbid with chronic pain, such as fatigue [24]. Furthermore, COVID-19 infection could even exacerbate their symptoms [9]. It is also possible that people with persistent pain due to COVID-19 infection are more likely to have other symptoms. Nevertheless, it is clear that people with persistent pain experience more-persistent symptoms that are common in “long COVID”, and higher levels of their associated burdens, such as depressive symptoms, anxiety, and insomnia, as observed in this study.

Overall, persistent symptoms showed a stronger association with psychological inflexibility compared with psychological flexibility. It is possible that people are more likely to be aware of their negative experiences, thus being better at reporting on processes related to these experiences, such as psychological inflexibility. It is also possible that the psychological inflexibility subscales can better assess related experiences. It is notable that some psychological flexibility processes that are historically associated with health and well-being, such as acceptance and experiential avoidance, did not show any association with general mental health in this study [25]. This pattern was also observed in a previous study investigating psychological flexibility using the MPFI in the context of COVID-19 [4]. Perhaps some of the subscales of the MPFI, developed based on the general population, are not the most suitable for capturing the experiences of specific clinical populations.

In line with previous studies, psychological flexibility appears to be associated with better general mental health, and psychological inflexibility with poorer general mental health, during COVID-19 in the general population [12,14] and among people with chronic pain [2,4]. Surprisingly, we only observed the moderating effect of psychological flexibility among people with persistent pain, and not in the expected direction. Specifically, the association between persistent symptoms and depression appeared stronger in participants with a higher level of psychological flexibility.

Again, it is possible that some of the key processes of psychological flexibility, such as acceptance, were not adequately assessed by the relevant subscale of the MPFI. It is also possible that participants with a relatively low level of psychological flexibility represent individuals with pre-existing chronic conditions, for whom the influence of persistent symptoms per se has become less dominant, and a more complex constellation of processes is involved in the deterioration of their health and well-being. In fact, we observed a less strong association between persistent physical symptoms and general mental health among people with persistent pain compared with those without pain. Similarly, this group of participants might represent people with pre-existing chronic pain, for whom the direct influence of symptoms has become less prominent.

Nevertheless, these findings suggest that the role of psychological (in)flexibility in the relationship between persistent symptoms and general mental health does not seem to differ substantially between people without pain and those with pain. Psychological inflexibility may be a useful process to target in treatment to improve general mental health in people with “long COVID”. Treatments that aim to reduce psychological inflexibility, such as ACT, can potentially help reduce the impact of persistent symptoms on health and well-being. In fact, ACT has been examined in people who were distressed during the pandemic and showed great potential in improving general mental health [17]. When developing and delivering interventions for people with “long COVID”, it is important to identify subgroups of patients with differing PF profiles and perhaps different pain profiles, so the intervention can target the most relevant processes for each subgroup of patients and optimise the treatment effect.

### 4.2. Limitations

This study had limitations. First and foremost, we did not collect data on the onset of participants’ persistent pain in relation to COVID-19 infection. Therefore, it was not possible to differentiate participants with pre-existing persistent pain from those who developed persistent pain due to COVID-19 infection. Nevertheless, the implications and challenges that come with living with persistent symptoms could be similar for all participants with persistent pain. Relatedly, data on participants’ chronic pain conditions were not collected, which hindered more fine-grained analyses of the implications of persistent symptoms for people with chronic pain.

Second, the sample size was relatively small, which may mean there was a limited representativeness of the sample and generalisability of the findings. Nonetheless, about 80% of the participants were white, and half were women, which is broadly consistent with the demographics of the UK population [26]. Studies using larger samples with a definitive “long COVID” diagnosis may help further understand the impact of persistent symptoms due to COVID-19 infection. Relatedly, the relatively small sample size may have limited the power of the analyses. Third, the sample sizes for people with and without pain were unequal. Although the unequal sample sizes were statistically adjusted when people with and without pain were statistically compared, the robustness of the comparison between these two sub-samples was more or less limited.

Next, the relatively small sample size limited our ability to comprehensively investigate the moderating role of each psychological flexibility and psychological inflexibility process, which would have provided a more fine-grained understanding of the role of these processes. Lastly, the cross-sectional design of this study did not allow for the inference of causal relations. Larger-scale, longitudinal studies are needed to examine the moderating role of psychological flexibility and psychological inflexibility processes in the impact of long COVID on general mental health.

### 4.3. Conclusions

Conditions with persistent symptoms, such as “long COVID”, can be particularly challenging for people with persistent pain. They experience higher burdens of persistent physical symptoms, poorer general mental health, and are less able to adapt to situational demands. Treatments that aim to reduce psychological inflexibility, such as ACT, can potentially help reduce the impact of persistent symptoms on health and well-being in people with “long COVID” symptoms. Further studies that include longitudinal designs are warranted to examine the moderating role of psychological flexibility and psychological inflexibility processes in the impact of “long COVID” on general mental health.

## Figures and Tables

**Table 1:** Background information of participants with and without pain.

Variable	Subcategory	n (%) or Mean (SD)	
		Pain	No pain
Gender	Women	27 (38.6)	64 (48.1)
	Men	42 (60)	69 (51.9)
	Other	1 (1.4)	0
Age		42.28 (40)	37.86 (14.06)
Ethnicity	White	57 (81.4)	114 (85.7)
	Black	5 (7.1)	13 (9.8)
	Mixed	5 (7.1)	2 (1.5)
	Other	3 (4.3)	1 (0.8)
	Asian	None	3 (2.3)
Years of education		14.71 (2.91)	15.44 (3.16)
Living area	Suburbs	49 (70)	57 (42.8)
	City	9 (12.9)	63 (47.4)
	Countryside	12 (17.1)	13 (9.8)
Working status	Working full-time	34 (48.6)	66 (49.6)
	Working part-time	15 (21.4)	36 (27.1)
	Unemployed	6 (8.6)	7 (5.3)
	Retired	4 (5.7)	7 (5.3)
	Sick leave	4 (5.7)	2 (1.5)
	Student	3 (4.3)	12 (9.0)
	Unpaid work (e.g., volunteer, carer, and homemaker)	2 (2.9)	3 (2.3)
	Parental leave	2 (2.9)	0
Work change due to COVID-19	Reduced working hours or workdays	26 (37.1)	53 (39.8)
	No change	16 (22.9)	45 (33.8)
	Taking sick leave	10 (14.3)	4 (3)
	Lost job	7 (10)	9 (6.8)
	Reduced salary	6 (8.6)	10 (7.5)
	Changed roles or responsibilities	5 (7.1)	12 (9)
Healthcare worker for COVID-19 patients	No	51 (72.9)	129 (97)
	Yes	18 (25.7)	4 (3)
Economic status	Average	32 (45.7)	73 (54.9)
	Below average	20 (28.6)	25 (18.8)
	Above average	9 (12.9)	27 (20.3)
	Much below average	6 (8.6)	5 (3.8)
	Much above average	3 (4.3)	2 (1.5)
Relationship status	Single	25 (35.7)	49 (36.8)
	Married	20 (28.6)	55 (41.4)
	In a relationship—living apart	12 (17.1)	9 (6.8)
	In a relationship—cohabitation	9 (12.9)	10 (7.5)
	Separated/divorced/widowed	4 (5.8)	7 (5.3)
Children under 18 years old	None	51 (72.9)	86 (64.7)
	One	9 (12.9)	29 (21.8)
	More than one	9 (12.9)	16 (12)
Number of people in household	1	16 (22.9)	36 (27.1)
	2	18 (25.7)	26 (19.5)
	3	11 (15.7)	37 (27.8)
	4	12 (17.1)	19 (14.3)
	More than 4	8 (11.4)	12 (9)
Having one or more persistent symptoms		70 (100)	81 (60.9)
Pain rating: 0–10		5.11 (2.20)	
Pain site	Chest	21 (30)	
	Lower back	20 (28.6)	
	Lower limbs	18 (25.7)	
	Upper shoulder or upper limbs	18 (25.7)	
	Head or face	17 (24.3)	
	Whole body	16 (22.9)	
	Abdomen	14 (20)	
	Neck	14 (20)	
	Pelvic region	10 (14.3)	
	Other	5 (7.1)	
		
		All participants n (%)	
Painful persistent physical symptoms	Joint pain	60 (29.4)	
	Headache	51 (25.1)	
	Chest pain	44 (21.6)	
	Pins and needles in the limbs	34 (16.7)	
	Sore throat	28 (13.8)	

**Table 2:** Mean and n for general mental health variables, PF variables, and total persistent symptoms, and *t*-test results.

Variables	Persistent Pain	n	Mean	SD	*t*	df	*p*	*d*
Depressive symptoms	Yes	70	14.90	5.85	8.33	201	<0.001	1.23
	No	133	7.62	5.95				
Anxiety	Yes	70	11.39	5.63	7.18	201	<0.001	1.01
	No	133	5.83	5.02				
Insomnia	Yes	70	14.26	6.80	6.22	201	<0.001	0.92
	No	133	8.54	5.91				
Psychological flexibility	Yes	70	3.34	0.85	1.14	201	0.255	0.17
	No	133	3.5	0.96				
Psychological inflexibility	Yes	66	3.31	0.83	2.86	189	0.005	0.44
	No	125	2.95	0.83				
Total persistent symptoms	Yes	70	9.57	6.12	7.34	105.38	<0.001	1.21
	No	133	3.55	4.28				

**Table 3:** Correlations between psychological flexibility and psychological inflexibility, general mental health, and total persistent physical symptoms among people with persistent pain and those without pain.

	Participants With Persistent Pain				Participants Without Persistent Pain			
	Depression	Anxiety	Insomnia	Persistent Physical Symptoms	Depression	Anxiety	Insomnia	Persistent Physical Symptoms
Persistent physical symptoms	0.38 **	0.26 *	0.21 **		0.45 ***	0.31 ***	0.52 ***	
Total psychological flexibility	−0.31 **	−0.22	−0.13	−0.22	−0.20 *	−0.23 **	−0.15	0.11
Total psychological inflexibility	0.63 ***	0.63 ***	0.46 ***	−0.003	0.51 ***	0.54 ***	0.43 ***	0.19 *
Acceptance	−0.22	−0.10	−0.13	−0.31 **	0.002	0.01	0.03	0.11
Present moment awareness	−0.06	0.01	0.05	−0.06	−0.11	−0.11	−0.08	0.15
Self-as-context	−0.29 *	−0.23	−0.12	−0.09	−0.22 *	−0.25 **	−0.16	0.07
Defusion	−0.37 **	−0.37 **	−0.14	−0.24 *	−0.29 ***	−0.36 ***	−0.29 ***	−0.01
Values	−0.18	−0.08	−0.07	−0.16	−0.14	−0.16	−0.09	0.15
Committed action	−0.39 ***	−0.31 **	−0.21	−0.24 *	−0.25 **	−0.28 **	−0.17	0.10
Experiential avoidance	0.14	0.04	0.17	−0.05	0.11	0.06	0.07	0.16
Lack of contact with present moment	0.28 *	0.30 *	0.27 *	−0.05	0.27 **	0.29 ***	0.21 *	0.05
Self-as-content	0.51 ***	0.64 ***	0.39 **	−0.11	0.43 ***	0.49 ***	0.33 ***	0.13
Fusion	0.67 ***	0.71 ***	0.46 ***	0.13	0.55 ***	0.60 ***	0.50 ***	0.28 **
Lack of contact with values	0.54 ***	0.47 ***	0.42 ***	0.04	0.47 ***	0.51 ***	0.41 ***	0.10
Inaction	0.69 ***	0.67 ***	0.41 ***	0.02	0.56 ***	0.60 ***	0.50 ***	0.17

Note: * *p* < 0.05; ** *p* < 0.01; and *** *p* < 0.001. n = 66–70 for participants with persistent pain sub-sample; n = 125–133 for participants without persistent pain sub-sample. When Bonferroni correction was applied (critical a = 0.025), persistent symptoms were not significantly correlated with anxiety anymore among people with persistent pain.

**Table 4:** Conditional effect of total persistent physical symptoms on depression at levels of psychological flexibility.

Psychological Flexibility	Estimate/Effect/Path Coefficient	*p*
Low	0.11	0.55
Medium	0.45	<0.01
High	0.75	<0.001

Note: When Bonferroni correction was applied (critical a = 0.025), significance remained for medium and high levels of psychological flexibility.

## Data Availability

The data that support the findings of this study will be openly available in the UK Data Service.
